# Association analysis between the interaction of RAS family genes mutations and papillary thyroid carcinoma in the Han Chinese population

**DOI:** 10.7150/ijms.50026

**Published:** 2021-01-01

**Authors:** Mengdi Jin, Zhijun Li, Yaoyao Sun, Mingyuan Zhang, Xin Chen, Hongguang Zhao, Qiong Yu

**Affiliations:** 1 Nuclear Medicine Department, First Hospital of Jilin University, Changchun 130021, China.; 2 Department of Epidemiology and Biostatistics, School of Public Health, Jilin University, Changchun 130021, China.

**Keywords:** PTC, RAS family genes, gene-gene interaction, SNP

## Abstract

Papillary thyroid carcinoma (PTC) is the major subtype of thyroid cancer, accounting for 75%-85% of all thyroid malignancies. This study aimed to identify the association between the interactions of single nucleotide polymorphisms (SNPs) in RAS family genes and PTC in the Han Chinese population, to provide clues to the pathogenesis and potential therapeutic targets for PTC. Hap Map and NCBI-db SNP databases were used to retrieve SNPs. Haploview 4.2 software was used to filter SNPs based on specific parameters, six SNPs of RAS gene (KRAS-rs12427141, KRAS-rs712, KRAS-rs7315339, HRAS-rs12628, NRAS-rs14804 and NRAS-rs2273267) were genotyped by matrix-assisted laser desorption/ionization time of flight mass spectrometry (MALDI-TOF-MS) in 673 PTC patients and 657 healthy controls, the interactive effect was evaluated by crossover analysis, logistic regression and GMDR software.

We found that genetic mutation in rs712 have significant associations with PTC risk after Bonferroni correction (*p*<0.001). The interaction between KRAS-rs12427141 and HRAS-rs12628 increased the risk of PTC (U=-2.119, *p*<0.05), the interaction between KRAS-rs2273267 and HRAS-rs7315339 reduced the risk of PTC (U=2.195, *p*<0.05). GMDR analysis showed that the two-factor model (KRAS-rs712, NRAS-rs2273267) was the best (*p*=0.0107). Summarily, there are PTC-related interactions between RAS family genes polymorphisms in the Han Chinese population.

## Introduction

Thyroid cancer is the most common malignant tumor of the endocrine system. Over the past 20 years, the incidence of thyroid cancer has increased dramatically worldwide 1. In China, the changes in the incidence of thyroid cancer between 1988 and 2009 were from 1.78 to 6.56 per 100, 000 individuals 2. Some believe that the improvement of socio-economic status, access to better health care, and the use of thyroid imaging led to the diagnoses of small, subclinical and stationary tumors with less impact on patients' has increased. However, epidemiological and clinical data suggests that a true increase in thyroid cancer cases has occurred 1.

It is worth noting that most of the increase in thyroid cancer diagnosis is due to the increase in papillary thyroid cancer (PTC), which accounts for 75%-85% of all thyroid malignancies 3. PTC is a multifactorial disease affected by both genetic and environmental factors, such as radiation exposure, iodine or industrial compound intake 4. However, its molecular mechanisms are still unclear.

Several studies have reported that the molecular pathogenesis of PTC is related to chromosomal rearrangement and point mutation of genes in the MAPK pathway 5. The protein encoded by the RAS is a very important signaling factor in the MAPK pathway and performs pivotal roles in the development of many tumors, such as lung cancer, pancreatic cancer and colorectal cancer 6-9. Furthermore, mutated RAS also accounts for the second most common mutation detected in biopsies from thyroid nodules which often results in malignancy 10. The oncogene RAS of the mammalian has three members, namely HRAS, KRAS and NRAS. They are all composed of four exons, located on the short arms of human chromosomes 11, 12, and 1, respectively 11. Their coded product is P21 protein, which plays an important role in cell growth, proliferation, and differentiation. They are related in structure and function.

The single nucleotide polymorphisms (SNPs) in the carcinogenesis-related genes might provide us a potential method to predict the risk and the prognosis of cancer. In our previous research, we found that variants in KRAS-rs712, KRAS-rs12427141 increased the risk of PTC 12. Considering the biological interaction between HRAS, NRAS, and KRAS, we sought to explore the relationship between the interaction of RAS family genes mutations and PTC in a case-control study, which has not been explored before.

In the genetic interaction analysis, there are three commonly used methods. The first one is logistic regression, it is a parameter estimation method, the existence and the effect of interaction can be evaluated by the regression coefficient β_3_ of the product term (Y = β_0_ + β_1_G_1_ + β_2_G_2_ + β_3_G_1_ × G_2_) 13. Du et al. conducted a multivariate logistic regression to analyze the impact of the interaction between miRNA-122 binding site polymorphisms and hepatitis B virus mutations on the risk of liver cancer 14. However, when processing high-dimensional data, the number of observations relative to the independent variables is too small to estimate parameters accurately. In this case, non-parametric methods are required to be combined with them. Multifactor Dimensionality Reduction (MDR) is a widely used non-parametric method for analyzing interactions and was first proposed by Ritchie et al 15. Generalized Multifactor Dimensionality Reduction (GMDR) is an extension of MDR, which also called score-based MDR. It follows the same algorithm framework, but substitutes the score values for the frequency, to evaluate the classification and prediction errors^16^. The basic idea is to transform the interaction effect between multiple SNPs to form a one-dimensional two-level variable, labeled as high-risk or low-risk, so it is easier to analyze the higher-order interaction 17, 18.

The third one is crossover analysis. Its results are more intuitive, and it can also make up for the fact that logistic regression cannot calculate the interaction under the additive model 19. Therefore, the three methods in this study complemented each other to explore the relationship between the interactions of the six SNPs on RAS family genes and the susceptibility to PTC, attempted to provide hopeful potential clues for the prevention, diagnosis, or treatment of PTC.

## Material and methods

### Subjects

673 patients with papillary thyroid cancer in China-Japan Union Hospital of Jilin University, China, from January 2012 to December 2014 were recruited as a case group. Each patient was diagnosed based on the Revised American Thyroid Association Management Guidelines and clinical-pathological sectioning. Patients with other cancer diagnoses were excluded. 657 healthy individuals without thyroid-related diseases from a cross-sectional survey of chronic diseases and risk factors among adults in Jilin Province from July to August 2012 were enrolled as the control group. Subjects with metabolic syndrome, thyroid-related diseases, other malignancy or mental disorders were excluded. Peripheral blood was provided by each participant for genotyping. The study was approved by the Ethics Committee of the School of Public Health, Jilin University, and written informed consent was obtained from all participants.

### SNP selection

Hap Map and NCBI-db SNP databases were used to retrieve KRAS, HRAS, and NRAS SNPs. Haploview 4.2 software was used to filter tag SNPs with r^2^>0.8, MAF (minor allele frequency)>10%, D'= 1, and CHB (Han population in northern China) as parameters. SNPs are preferably sites of missense mutations, introns in the regulatory region are the second choice. A total of 6 SNPs was selected in this study, rs712, rs7315339, and rs12427141 of the KRAS gene, rs12628 of the HRAS gene, and rs2273267 and rs14804 of the NRAS gene.

### DNA extraction and genotyping

5 ml of peripheral blood obtained from each participant was stored in EDTA-containing tubes. Genomic DNA was extracted according to the instruction of the ClotBlood DNA Kit (Cwbio, Beijing, China). The DNA concentration was measured using the ultraviolet spectrophotometer (Beckman, USA). Genotypes were detected by iPLEX Gold reagent set (Sequenom, San Diego, CA, USA). The standard polymerase chain reaction (PCR) was performed and PCR products were assayed by matrix-assisted laser desorption/ionization time of flight mass spectrometry (MALDI-TOF-MS). The PCR primers were designed by Assay Designer 3.1 (Table 1). Samples that failed genotyping were removed from the analyses. The internal blinded quality control of the samples showed 97% consistency.

### Statistical analysis

The Student's t-test or χ^2^ test was used to analyze age and gender differences between the PTC and NC groups. The Hardy-Weinberg equilibrium test was applied to each SNP in both groups. The associations of SNPs and PTC were tested using the Fisher exact test or χ^2^ test. The interaction between SNPs in different genes was evaluated by logistic regression, GMDR software (v0.7) and crossover analysis. All statistical tests were two-tailed. *p*<0.05 was considered statistically significant. For the Bonferroni correction on the *p*-values, we used *p*=0.05/6=0.0083 as a threshold of significance.

## Results

### Subject characteristics and Hardy-Weinberg equilibrium

A total of 1330 subjects were recruited in this study. No statistically significant difference was observed in gender and age between PTC patients (male/female=140/533, mean age=44.17±9.17) and healthy controls (male/female=165/492, mean age=44.46±8.95) (all *p* >0.05). The genotype distributions of the six SNPs were consistent with the Hardy-Weinberg equilibrium in both case and control groups (*p* >0.05).

### The Allele and Genotype Analysis

The genotype distribution and allele frequencies of rs12427141 and rs712 located in the KRAS gene were significantly different between the PTC group and NC group (rs12427141: genotype *P*=0.009, allele *P*=0.005; rs712: genotype *P*<0.001, allele *P*=0.001) (Table 2). However, the difference in genotype distribution of rs12427141 became insignificant after Bonferroni correction. Furthermore, we found significantly elevated risk in the KRAS-rs1242714 homozygous variant (OR_AA/GG_=3.380, 95%CI=1.229-9.297), the KRAS-rs1242714 heterozygous variant (OR_GA/GG_=1.291, 95%CI=1.002-1.663), and the KRAS-rs712 heterozygous variant (OR_GT/GG_=1.614, 95%CI=1.274-2.046). The allelic or genotypic association between the other four SNPs and PTC was not found (all, *p*>0.05).

### Analysis of gene-gene interaction

The additive model calculated by crossover analysis revealed that there was a significant PTC-related positive interaction between KRAS-rs12427141 and HRAS-rs12628 (U=-2.119, *p*<0.05), the presence of both variation might increase the risk of PTC (S=0.121), and the effect of interaction on PTC susceptibility is 0.709 times that of other unknown factors (RERI=-0.709), the effect of interaction accounted for 64.6% in PTC when both mutations exist (*AP*=-0.646). Similarly, there was a negative interaction between KRAS-rs2273267 and HRAS-rs7315339 (U=2.195, *p*<0.05, *S*=-0.195), the effect of the interaction was 0.643 times that of other unknown factors, and the interaction of them accounts for 71.8% in PTC (Table 3). However, there was no significant PTC-related interaction between the 6 SNPs under the multiplication model conducted by logistic regression (Table 4).

**Figure 1:**
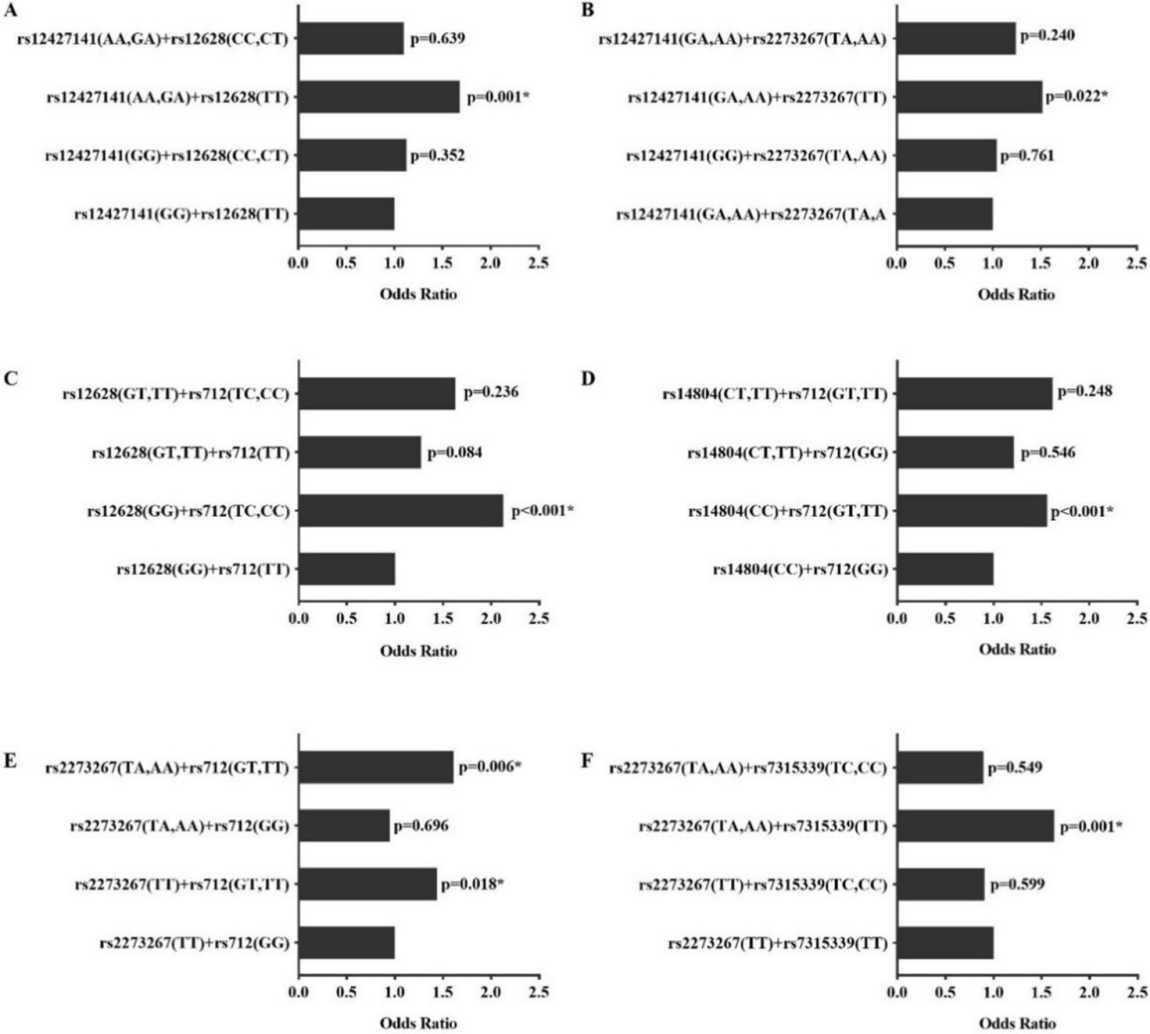
Interactions between different genotype combinations. The *p* value is the result compared with wild type homozygote, *, *p*<0.05.

In addition to the interactions between SNPs, different genotype combinations also exhibited different effects on PTC (Figure 1). Subjects with genotype combinations AA/GA on rs12427141and TT on rs12628 had a higher risk of PTC (OR = 1.681, 95% CI =1.220-2.316, *p*=0.001) compared with other genotypes. Similarly, the genotype combinations rs12427141 (AA/ GA) and rs227326 (TT), rs12628 (GG) and rs712 (TC/CC), rs14804 (CC) and rs712 (GT/TT), rs2273267 (TA/AA/TT) and rs712 (GG/TT), rs2273267 (TA/AA) and rs7315339 (TT) increased the risk of PTC compared with the wild type (OR>1, *p* <0.05).

**Figure 2:**
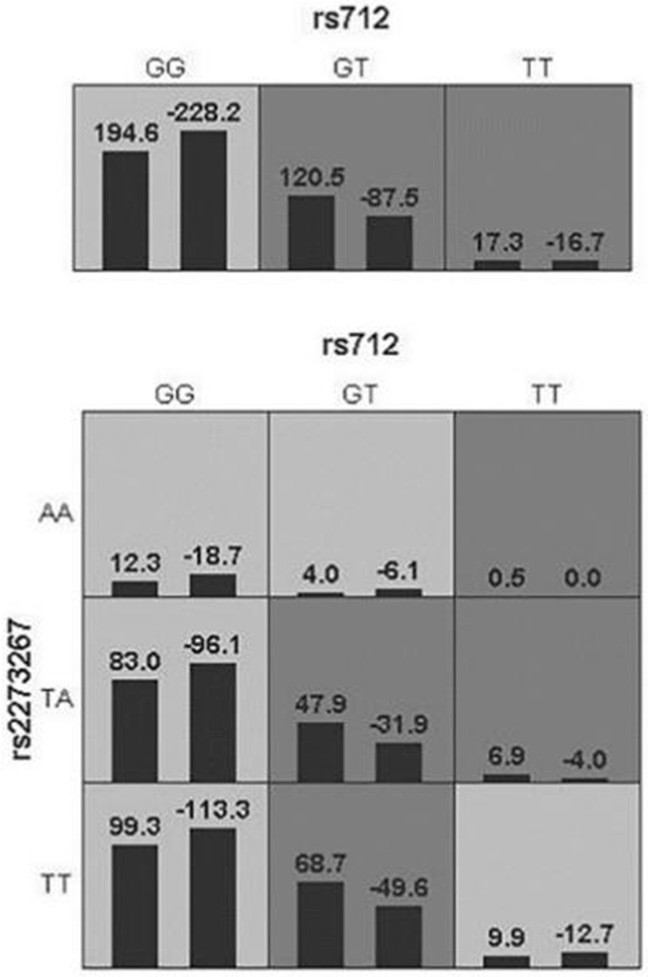
A combined model of interaction between RAS family genes. The left bar of the cell represents the case group, the right one is the control group. High risk and low risk are represented by dark gray and light gray respectively, a blank cell indicates no effect or no such genotype in the population.

The GMDR software finally screened out a single-factor model (KRAS-rs712) and a two-factor model (KRAS-rs712, NRAS-rs2273267). The two-factor model was better with an accuracy of 54.59%, 8 for cross-validation Consistency, and *p*=0.0107 for Sign Test (Table 5). The ring diagram visually reflected the interaction between SNPs through the distribution of high-risk and low-risk differences (Figure 2).

## Discussion

It has been confirmed by academia that papillary thyroid carcinoma is a polygenic disease with complex etiology. As a high-quality potential biomarker for tumors, single nucleotide polymorphisms (SNPs) have been reported in many studies on the association with PTC, including rs965513, rs944289, rs1801516, rs2439302, rs10136427, rs7267944, rs2910264, rs966423, etc. 20-26. However, emerging research has prompted us that most genes do not work independently, but often the superposition of the genetic susceptibility of multiple micro-effect genes, combined with the influence of environmental factors, finally contributed to the occurrence of disease. Some researchers have found that there are interactions between genes in many complex genetic diseases 27, 28. Gene interaction refers to the clinical phenotypes not only depend on one pair of alleles but also affected by other non-allelic genes; also called cumulative effects, inhibitory effects, activation effects, ectopic dominant, modification, etc.

The RAS family genes are a class of proto-oncogenes that are very conservative during biological evolution, including KRAS, HRAS, and NRAS. They are closely related in function and structure, and both encode G proteins. The G protein can be combined with guanylate, it is inactivated when combined with GDP and activated with GTP. Therefore, RAS gene family can be regarded as a molecular switch, switching between two different conformations 29. Elda Grabocka et al confirmed that cancer cells with KRAS mutations require wild-type HRAS to proliferate through mitosis, and the knockdown of wild-type HRAS and NRAS can enhance the DNA damage of cancer cells with KRAS mutations 30. In view of the mutual influence of KRAS, NRAS, and HRAS in the performance of certain functions, we studied the effect of the interaction between RAS family genes on the susceptibility to PTC in the Han population. Six SNPs were selected with Haploview 4.2 according to the screening criteria, and the interactions of SNPs on different genes were calculated by three complementary approaches.

Our data suggested that the interaction between KRAS-rs12427141 and HRAS-rs12628 increased the risk of PTC, and the interaction between NRAS-rs2273267 and KRAS-rs7315339 reduced the risk of PTC. The interaction between KRAS-rs712 and NRAS-rs2273267 calculated by GMDR might also participate in the pathogenesis of PTC (*p* = 0.0107). It is worth mentioning that there are two interactive hypothesis testing methods for crossover analysis. One is to use the Excel calculation table constructed by Tomas Andersson, which calculates the 95% confidence interval of *RER*I 31. Another is to calculate the statistic U according to the formula proposed by Rothman et al and look up the boundary value table 32. We adopted the latter, that's why the *p*-value is a range rather than an exact value. However, statistical interactions do not necessarily indicate that there must be interactions in biology. How do they interact? Are these interactions merely involved in the normal pathogenesis of PTC or caused PTC? This study cannot explain yet, and we would continue to search for answers in future studies.

This study has some limitations. First, since the allele and genotype frequencies of SNPs strongly depend on ethnic background, large-scale studies with different ethnicities are still needed to validate our findings and explore the underlying mechanism of RAS gene family interactions in PTC development. Secondly, environmental factors such as iodine intake, radiation, and chemical products are also major driving forces for PTC development. In the next step, consideration should be given to incorporating the environmental factors in a more comprehensive interaction study with genes. Despite these limitations, this study is an extension of the previous work. To our knowledge, this is the first study to provide evidence for the association between PTC susceptibility and the interaction between RAS family genes, and parametric and non-parametric methods were used to complement each other to explore more possible interactions.

Clinical studies targeting RAS protein have been found to be highly successful for progression-free survival in patients with thyroid cancer 33, and the exploration of the SNPs interaction in complex diseases such as PTC might make a breakthrough in the pathogenesis of the disease, thereby making the way to intervene in the process of tumor development no longer single.

## Conclusions

Genetic variants in rs712 have significant associations with PTC risk. The interaction between KRAS-rs12427141 and HRAS-rs12628 increased the risk of PTC, and the interaction between NRAS-rs2273267 and KRAS-rs7315339 reduced the risk of PTC. The interaction between KRAS-rs712 and NRAS-rs2273267 calculated by GMDR might also participate in the pathogenesis of PTC. According to our data, there are PTC-related interactions between RAS family genes polymorphisms, which might provide clues for the multi-target treatment of papillary thyroid carcinoma.

## Figures and Tables

**Table 1:** Primers of RAS genetic polymorphisms for PCR amplification

SNP	Alleles	Primer sequence	HWE
rs12427141	G>A	F: 5'-ACGTTGGATGCAAGACAGAGTTTAATACGGG-3'	1.000
		R: 5'-ACGTTGGATGGGGACATAGTAACTACCCAG-3'	
rs712	G>T	F: 5'-ACGTTGGATGCCAAAACTCTGGGAATACTG-3'	0.963
		R: 5'-ACGTTGGATGGCAGACTGTTAGCTTTTACC-3'	
rs7315339	T>C	F: 5'-ACGTTGGATGGAAAAGCTAGTAAGGAGTGG-3'	0.075
		R: 5'-ACGTTGGATGGATGGTAGATGGTAATTCAG-3'	
rs12628	T>C	F:5'-ACGTTGGATGATATAAGCTGGTGGTGGTGG -3'	0.472
		R: 5'-ACGTTGGATGATAGTGGGGTCGTATTCGTC -3'	
rs2273267	T>A	F: 5'-ACGTTGGATGTATTGAAACGTCCCGTGTGG -3'	0.273
		R: 5'-ACGTTGGATGCACGTGGGCCTCCGAACCA -3'	
rs14804	C>T	F: 5'-ACGTTGGATGGAAAAAAGTGGAGATTAGG -3'	0.602
		R: 5'-ACGTTGGATGGCTTTCCCAGGAGAAAGATG -3'	

Abbreviations: F, forward; R, reverse; HWE, Hardy-Weinberg equilibrium.

**Table 2:** The genotype and allele frequencies of SNPs in the PTC and NC groups

SNPs	Group	Genotypic association (%)					Allelic association (%)			
		1/1	1/2	2/2	χ^2^	*p*	1	2	χ^2^	*p*
rs12427141 (G>A)	PTC	481 (71.5)	176 (26.2)	16 (2.4)	9.508	0.009	1138 (84.5)	208 (15.5)	7.882	**0.005**
	Control	508 (77.3)	144 (21.9)	5 (0.8)			1160 (88.3)	154 (11.7)		
rs712 (G>T)	PTC	394 (58.5)	244 (36.6)	35 (5.2)	15.802	<0.001	1032 (76.7)	314 (23.3)	10.666	**0.001**
	Control	451 (68.6)	173 (26.3)	33 (5.0)			1075 (81.8)	239 (18.2)		
rs7315339 (T>C)	PTC	532 (79.0)	140 (20.8)	1 (0.1)	-	0.228^a^	1204 (89.5)	142 (10.5)	0.807	0.369
	Control	509 (77.5)	143 (21.8)	5 (0.8)			1161 (88.4)	153 (11.6)		
rs12628 (T>C)	PTC	380 (56.5)	262 (38.9)	31 (4.6)	0.944	0.624	1022 (75.9)	324 (24.1)	0.390	0.532
	Control	365 (55.6)	254 (38.7)	38 (5.8)			984 (74.9)	330 (25.1)		
rs14804 (C>T)	PTC	638 (94.8)	35 (5.2)	0 (0)	-	0.483^a^	1311 (97.4)	35 (2.6)	0.087	0.769
	Control	627 (95.4)	29 (4.4)	1 (0.2)			1283 (97.6)	31 (2.4)		
rs2273267 (T>A)	PTC	360 (53.5)	279 (41.5)	34 (5.1)	3.358	0.187	999 (74.2)	347 (25.8)	0.810	0.368
	Control	347 (52.8)	261 (39.7)	49 (7.5)			955 (72.7)	359 (27.3)		

Notes: Data were presented as number (proportion). *P* values were calculated by χ^2^ test or Fisher's exact test. a, Fisher's exact test. 1, reference allele; 2, variant allele; 1/1, homozygous wild; 1/2, heterozygous; 2/2, homozygous mutational. Abbreviations: PTC, papillary thyroid carcinoma.

**Table 3:** Crossover analysis of the PTC-related interactions between SNPs of RAS genes

Loci combinations		PTC	Control	OR (95%CI)	*S*	*RERI*	*AP*	*P*
rs12427141* rs12628								
+	+	61	62	1.098 (0.742-1.627)	0.121	-0.709	-0.646	**<0.05**
+	-	131	87	1.681 (1.220-2.316)				
-	+	232	230	1.126 (0.877-1.446)				
-	-	249	278	1				
rs12427141*rs14804								
+	+	12	5	2.531 (0.885-7.240)	17.398	1.443	0.507	>0.05
+	-	193	182	1.118 (0.880-1.421)				
-	+	23	25	0.970 (0.543-1.734)				
-	-	458	483	1				
rs12427141*rs2273267								
+	+	83	72	1.240 (0.866-1.776)	0.430	-0.318	-0.256	>0.05
+	-	103	73	1.518 (1.074-2.144)				
-	+	230	238	1.040 (0.810-1.334)				
-	-	251	270	1				
rs12628*rs14804								
+	+	14	11	1.227 (0.549-2.739)	10.318	0.205	0.167	>0.05
+	-	279	281	0.957 (0.766-1.195)				
-	+	21	19	1.065 (0.563-2.016)				
-	-	359	346	1				
rs12628*rs2273267								
+	+	101	99	0.969 (0.689-1.363)	0.564	0.024	0.025	>0.05
+	-	161	158	0.968 (0.720-1.302)				
-	+	181	176	0.977 (0.733-1.302)				
-	-	199	189	1				
rs12628*rs712								
+	+	14	11	1.626 (0.723-3.657)	0.448	-0.770	-0.474	>0.05
+	-	192	193	1.271 (0.968-1.667)				
-	+	178	107	2.125 (1.570-2.875)				
-	-	202	258	1				
rs12628*rs7315339								
+	+	67	82	0.798 (0.557-1.144)	-1.365	-0.35	-0.439	>0.05
+	-	226	210	1.052 (0.822-1.345)				
-	+	74	66	1.096 (0.758-1.583)				
-	-	306	299	1				
rs14804*rs712								
+	+	14	10	1.618 (0.710-3.685)	0.797	-0.157	-0.097	>0.05
+	-	21	20	1.213 (0.648-2.273)				
-	+	265	196	1.562 (1.240-1.968)				
-	-	373	431	1				
rs14804*rs7315339								
+	+	4	6	0.645 (0.181-2.301)	-1.929	-0.539	-0.836	>0.05
+	-	31	24	1.250 (0.723-2.162)				
-	+	137	142	0.934 (0.716-1.218)				
-	-	501	485	1				
rs2273267*rs712								
+	+	120	83	1.611 (1.149-2.260)	1.571	0.222	0.138	>0.05
+	-	193	227	0.948 (0.723-1.242)				
-	+	159	123	1.441 (1.064-1.950)				
-	-	201	224	1				
rs2273267*rs7315339								
+	+	72	76	0.895 (0.623-1.286)	-0.195	-0.643	0.718	**<0.05**
+	-	171	99	1.632 (1.212-2.198)				
-	+	69	72	0.906 (0.626-1.310)				
-	-	291	275	1				

Notes: +, Mutant heterozygote and homozygous; -, Unmutated wild type. Abbreviations: PTC, papillary thyroid carcinoma; *S*, synergy index; *RERI*, relative excess risk due to interaction; *AP*: attributable proportion due to interaction.

**Table 4:** The PTC-related interactions between SNPs based on logistic regression

	B	S.E.	Wald	*p*	OR	95%CI
rs12427141*rs12628	0.207	0.184	1.264	0.262	1.230	0.857-1.765
rs12427141*rs14804	-0.652	0.503	1.679	0.195	0.521	0.194-1.397
rs12427141*rs2273267	0	0.167	0	0.999	1.000	0.721-1.389
rs12628*rs14804	-0.025	0.389	0.004	0.949	0.975	0.455-2.091
rs12628*rs2273267	0.162	0.135	1.445	0.229	1.176	0.903-1.532
rs12628*rs712	0.160	0.150	1.135	0.287	1.173	0.875-1.573
rs12628*rs7315339	0.337	0.173	3.801	0.051	1.400	0.998-1.965
rs14804*rs712	-0.107	0.397	0.072	0.789	0.899	0.413-1.958
rs14804*rs7315339	0.614	0.629	0.953	0.329	1.848	0.538-6.342
rs2273267*rs712	-0.255	0.151	2.878	0.090	0.775	0.577-1.040
rs2273267*rs7315339	0.160	0.173	0.859	0.354	1.174	0.837-1.646

Abbreviations: B, Regression coefficients; S.E., Standard error; Wald, Chi-square value; OR, Odds ratio.

**Table 5:** GMDR analysis of the interactions between the tag SNPs for PTC

Model	Tr-BA	Te-BA	Sign Test (*p*)	CVC
rs712	0.5509	0.5423	9 (0.0107)	10/10
rs712/rs2273267	0.5581	0.5459	9 (0.0107)	8/10

Abbreviations: Tr-BA, training balanced accuracy; Te-BA, testing balanced accuracy; CVC, cross validation consistency.

## References

[1] Vigneri R, Malandrino P, Vigneri P (2015). The changing epidemiology of thyroid cancer: why is incidence increasing?. Current opinion in oncology.

[2] Du L, Wang Y, Sun X, Li H, Geng X, Ge M (2018). Thyroid cancer: trends in incidence, mortality and clinical-pathological patterns in Zhejiang Province, Southeast China. BMC Cancer.

[3] Romei C, Elisei R (2012). RET/PTC Translocations and Clinico-Pathological Features in Human Papillary Thyroid Carcinoma. Front Endocrinol (Lausanne).

[4] Santos M, Azevedo T, Martins T, Rodrigues FJ, Lemos MC (2014). Association of RET genetic polymorphisms and haplotypes with papillary thyroid carcinoma in the Portuguese population: a case-control study. PLoS One.

[5] Myers MB, McKim KL, Parsons BL (2014). A subset of papillary thyroid carcinomas contain KRAS mutant subpopulations at levels above normal thyroid. Mol Carcinog.

[6] Shah S, Brock EJ, Ji K, Mattingly RR (2019). Ras and Rap1: A tale of two GTPases. Seminars in cancer biology.

[7] Lanfredini S, Thapa A, O'Neill E (2019). RAS in pancreatic cancer. Biochem Soc Trans.

[8] Yang L, Zhang H, Chen D, Ding P, Yuan Y, Zhang Y (2018). EGFR and Ras regulate DDX59 during lung cancer development. Gene.

[9] Yiu AJ, Yiu CY (2016). Biomarkers in Colorectal Cancer. Anticancer research.

[10] Bhaijee F, Nikiforov YE (2011). Molecular analysis of thyroid tumors. Endocrine pathology.

[11] Bos JL (1988). The ras gene family and human carcinogenesis. Mutat Res.

[12] Ning L, Rao W, Yu Y, Liu X, Pan Y, Ma Y (2016). Association between the KRAS Gene Polymorphisms and Papillary Thyroid Carcinoma in a Chinese Han Population. J Cancer.

[13] García-Magariños M, López-de-Ullibarri I, Cao R, Salas A (2009). Evaluating the ability of tree-based methods and logistic regression for the detection of SNP-SNP interaction. Ann Hum Genet.

[14] Du Y, Han X, Pu R, Xie J, Zhang Y, Cao G (2014). Association of miRNA-122-binding site polymorphism at the interleukin-1 α gene and its interaction with hepatitis B virus mutations with hepatocellular carcinoma risk. Frontiers of medicine.

[15] Ritchie MD, Hahn LW, Roodi N, Bailey LR, Dupont WD, Parl FF (2001). Multifactor-dimensionality reduction reveals high-order interactions among estrogen-metabolism genes in sporadic breast cancer. American journal of human genetics.

[16] Lou XY, Chen GB, Yan L, Ma JZ, Zhu J, Elston RC (2007). A generalized combinatorial approach for detecting gene-by-gene and gene-by-environment interactions with application to nicotine dependence. American journal of human genetics.

[17] Xu HM, Xu LF, Hou TT, Luo LF, Chen GB, Sun XW (2016). GMDR: Versatile Software for Detecting Gene-Gene and Gene-Environ- ment Interactions Underlying Complex Traits. Curr Genomics.

[18] Zhu Z, Tong X, Zhu Z, Liang M, Cui W, Su K (2013). Development of GMDR-GPU for gene-gene interaction analysis and its application to WTCCC GWAS data for type 2 diabetes. PLoS One.

[19] Wu YZ, Yang H, Zhang L, Zhang YQ, Liu L, Yi D (2012). Application of Crossover Analysis-logistic Regression in the Assessment of Gene- environmental Interactions for Colorectal Cancer. Asian Pac J Cancer Prev.

[20] Ai L, Liu X, Yao Y, Yu Y, Sun H, Yu Q (2014). Associations between rs965513/rs944289 and papillary thyroid carcinoma risk: a meta-analysis. Endocrine.

[21] Damiola F, Byrnes G, Moissonnier M, Pertesi M, Deltour I, Fillon A (2014). Contribution of ATM and FOXE1 (TTF2) to risk of papillary thyroid carcinoma in Belarusian children exposed to radiation. Int J Cancer.

[22] Figlioli G, Köhler A, Chen B, Elisei R, Romei C, Cipollini M (2014). Novel genome-wide association study-based candidate loci for differentiated thyroid cancer risk. J Clin Endocrinol Metab.

[23] Jendrzejewski J, He H, Radomska HS, Li W, Tomsic J, Liyanarachchi S (2012). The polymorphism rs944289 predisposes to papillary thyroid carcinoma through a large intergenic noncoding RNA gene of tumor suppressor type. Proc Natl Acad Sci U S A.

[24] Liyanarachchi S, Wojcicka A, Li W, Czetwertynska M, Stachlewska E, Nagy R (2013). Cumulative risk impact of five genetic variants associated with papillary thyroid carcinoma. Thyroid.

[25] Świerniak M, Wójcicka A, Czetwertyńska M, Długosińska J, Stachlewska E, Gierlikowski W (2016). Association between GWAS-Derived rs966423 Genetic Variant and Overall Mortality in Patients with Differentiated Thyroid Cancer. Clin Cancer Res.

[26] Ai L, Yu Y, Liu X, Wang C, Shi J, Sun H (2014). Are the SNPs of NKX2-1 associated with papillary thyroid carcinoma in the Han population of Northern China?. Frontiers of medicine.

[27] Wang L, Cheng F, Hu J, Wang H, Tan N, Li S (2018). Pathway-based gene-gene interaction network modelling to predict potential biomarkers of essential hypertension. Bio Systems.

[28] Stone S, Abkevich V, Russell DL, Riley R, Timms K, Tran T (2006). TBC1D1 is a candidate for a severe obesity gene and evidence for a gene/gene interaction in obesity predisposition. Hum Mol Genet.

[29] Wittinghofer A, Pai EF (1991). The structure of Ras protein: a model for a universal molecular switch. Trends in biochemical sciences.

[30] Grabocka E, Pylayeva-Gupta Y, Jones MJ, Lubkov V, Yemanaberhan E, Taylor L (2014). Wild-type H- and N-Ras promote mutant K-Ras-driven tumorigenesis by modulating the DNA damage response. Cancer Cell.

[31] Andersson T, Alfredsson L, Källberg H, Zdravkovic S, Ahlbom A (2005). Calculating measures of biological interaction. Eur J Epidemiol.

[32] Rothman KJ. Epidemiology: An Introduction, (2nd ed.). New York Ny Oxford University Press. 2012

[33] Hong DS, Sebti SM, Newman RA, Blaskovich MA, Ye L, Gagel RF (2009). Phase I trial of a combination of the multikinase inhibitor sorafenib and the farnesyltransferase inhibitor tipifarnib in advanced malignancies. Clin Cancer Res.

